# *Bacillus thuringiensis* subsp. *kurstaki* HD1 as a factory to synthesize alkali-labile ChiA74∆sp chitinase inclusions, Cry crystals and spores for applied use

**DOI:** 10.1186/1475-2859-13-15

**Published:** 2014-01-24

**Authors:** José Eleazar Barboza-Corona, Jorge Luis Delgadillo-Ángeles, José Cristóbal Castañeda-Ramírez, Uriel Eleazar Barboza-Pérez, Luz Edith Casados-Vázquez, Dennis K Bideshi, Ma Cristina del Rincón-Castro

**Affiliations:** 1 Universidad de Guanajuato Campus Irapuato-Salamanca, División Ciencias de la Vida, Posgrado en Biociencias, Irapuato, Guanajuato 36500, México; 2 Universidad de Guanajuato Campus Irapuato-Salamanca, División Ciencias de la Vida, Departamento de Alimentos, Irapuato, Guanajuato 36500, México; 3 Tecnológico de Monterrey Campus Querétaro, Epigmenio González 500 Fracc., San Pablo Querétaro, Querétaro 76130, México; 4 Department of Natural and Mathematical Sciences, California Baptist University, 8432 Magnolia Avenue, Riverside California 92504, USA; 5 Department of Entomology, University of California, Riverside California 92521, USA

**Keywords:** *Bacillus thuringiensis*, Endochitinase ChiA74, Cry proteins, Inclusion bodies

## Abstract

**Background:**

The endochitinase ChiA74 is a soluble secreted enzyme produced by *Bacillus thuringiensis* that synergizes the entomotoxigenecity of Cry proteins that accumulate as intracellular crystalline inclusion during sporulation. The purpose of this study was to produce alkaline-soluble ChiA74∆sp inclusions in *B. thuringiensis*, and to determine its effect on Cry crystal production, sporulation and toxicity to an important agronomical insect, *Manduca sexta*. To this end we deleted the secretion signal peptide-coding sequence of *chiA74* (i.e. *chiA74∆sp*) and expressed it under its native promoter (pEHchiA74∆sp) or strong chimeric sporulation-dependent *cytA-p*/STAB-SD promoter (pEBchiA74∆sp) in *Escherichia coli*, acrystalliferous *B. thuringiensis* (4Q7) and *B. thuringiensis* HD1.

**Results:**

Based on mRNA analyses, up to ~9-fold increase in expression of *chiA74∆sp* was observed using the *cytA-p*/STAB-SD promoter. ChiA74∆sp (~70 kDa) formed intracellular inclusions that frequently accumulated at the poles of cells. ChiA74∆sp inclusions were dissolved in alkali and reducing conditions, similar to Cry crystals, and retained its activity in a wide range of pH (5 to 9), but showed a drastic reduction (~70%) at pH 10. Chitinase activity of *E. coli-*pEHchiA74∆s*p* was *~*150 mU/mL, and in *E. coli-*pEBchiA74∆s*p*, 250 mU/mL. 4Q7-pEBchiA74∆sp and 4Q7-pEHchiA74∆sp had activities of ~127 mU/mL and ~41 mU/mL, respectively. The endochitinase activity in HD1-pEBchiA74∆sp increased 42x when compared to parental HD1 strain. HD1-pEBchiA74∆sp and HD1 harbored typical bipyramidal Cry inclusions, but crystals in the recombinant were ~30% smaller. Additionally, a 3x increase in the number of viable spores was observed in cultures of the recombinant strain when compared to HD1. Bioassays against first instar larvae of *M. sexta* with spore-crystals of HD1 or spore-crystal-ChiA74∆sp inclusions of HD1*-*pEBchiA74∆sp showed LC_50_s of 67.30 ng/cm^2^ and 41.45 ng/cm^2^, respectively.

**Conclusions:**

Alkali-labile ChiA74∆sp inclusion bodies can be synthesized in *E. coli* and *B. thuringiensis* strains. We demonstrated for the first time the applied utility of synthesis of ChiA74∆sp inclusions, Cry crystals and spores in the same sporangium of HD1, a strain used successfully worldwide to control economically significant lepidopteran pests of agriculture. Our findings will allow to us develop strategies to modify expression of ChiA74∆sp while maximizing Cry crystal synthesis in commercial strains of *B. thuringiensis*.

## Background

*Bacillus thuringiensis, B. sphaericus, Paenibacillus popilliae, Clostridium bifermentans* and *Brevibacillus laterosporus* are spore-forming bacteria that produce intracellular crystalline or non-crystalline inclusions, many of which are toxic to insect pests of agriculture and medically significant vectors of disease [1,2]. In particular, parasporal bodies of *B. thuringiensis* subsp. *kurstaki* (HD1) and *B. thuringiensis* subsp. *israelensis*, toxic to lepidopteran and dipteran larvae, respectively, are among the most successful bioinsecticides used worldwide [3,4], and are composed of a plethora of Cry (crystal) or Cyt (cytolytic) proteins that are synthesized and occluded during sporulation [5-8]. In addition to Cry and Cyt protoxins, *B. thuringiesis* also synthesizes a battery of soluble chitinolytic enzymes secreted during vegetative growth that hydrolyze environmental chitin substrates for use as carbon and nitrogen sources. Chitinases are generally produced at a markedly lower level than Cry and Cyt. As such, unlike Cry and Cyt, and perhaps disadvantageous to efficient commercial formulations, chitinases do not naturally accumulate as intracellular inclusions in bacterial cells [9-14].

From an applied perspective, chitinases could be a useful component of *B. thuringiensis*-based biopesticides as they could function to degrade chitin polymers present in the protective midgut peritrophic membrane of insect larvae. In fact, previous studies have demonstrated that the hydrolytic activity of chitinase synergizes the toxicological effects of Cry, presumably by enhancing binding of active toxin ligand to microvillar membrane receptors [11,13-15]. Interestingly, it has been shown that when the chitinase gene of *B. thuringiensis* strain 4.0718 lacking its secretion signal peptide coding sequence was expressed under sporulation-dependent promoters, spherical intracellular inclusion accrued, and when these purified inclusions were mixed with Cry1Ac, an ~1.5x increase in toxicity against *Spodoptera exigua* and *Helicoverpa armigera* was observed [11]. Similar studies using secreted soluble chitinases have also demonstrated that these enzymes enhance the toxicity of Cry1Ac. For example, the chitinase gene of *Nicotiana tabacum* expressed simultaneously with *cry1Ac* in an acrystalliferous strain of *B. thuringiensis* using the BtI-BtII promoters showed increases in chitinolytic activity (6x) and toxicity (18×) of the recombinant bacterium against *Helicoverpa armigera* Hubner [14]. These methods of expression of chitinase genes under sporulation-dependent promoters appear to be more robust compared to the synthesis of unstable chimeric protein composed of Chi255 and the C-terminal half (crystallization domain) of Cry1Ac [15].

Recently, we have reported an unprecedented ~300-fold increase in synthesis of ChiA74, an endochitinase native to *B. thuringiensis*, in a recombinant strain of *B. thuringiensis* HD73 [16] when *chiA74* was expressed under control of the strong chimeric promoters, *cytA-p/*STAB-SD, developed by Park et al. (1998) [17]. In this study, we used the wildtype promoter of *chiA74* or *cytA-p/*STAB-SD to express *chiA74* lacking the sequence coding for the secretion signal peptide (*chiA74*∆s*p*) in *Escherichia coli*, acrystalliferous *B. thuringiensis* subsp. *israelensis* strain 4Q7, and *B. thuringiensis* subsp. *kurstaki* HD1, a strain used successfully worldwide in agriculture as a biodegradable lepidopteran larvicide. Using this strategy, we were able to produce stable ChiA74∆sp inclusions in *E. coli,* and also, for the first time, intracellular ChiA74∆sp inclusion together with Cry crystals and spores in *B. thuringiensis* subsp. *kurstaki* HD1. We demonstrate the utility of the recombinant HD1 strain against larvae *Manduca sexta*. Our results lay a foundation for similar engineering of other commercial strains of *B. thuringiensis*.

## Results

### ChiA74Δsp accumulates as inclusion bodies *in Escherichia coli*

**Figure 1:**
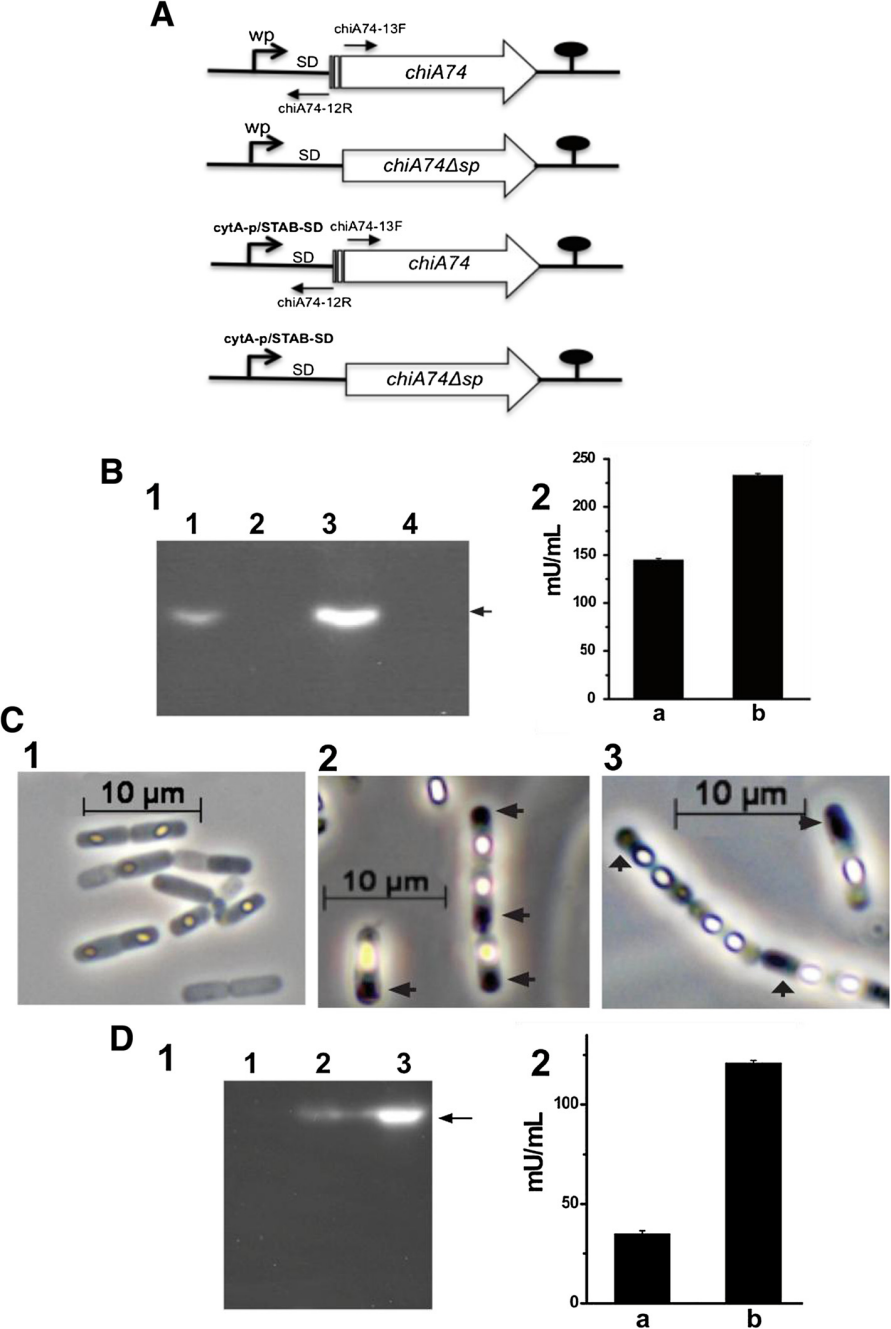
**Expression of ChiA74Δsp in *Escherichia coli *DH5α and *Bacillus thuringiensis *4Q7. (A)** Schematic illustration of the strategy used to delete the secretion signal peptide-encoding sequence (shown as a rectangle inside the open reading frame) of *chiA74* to generate *chiA74∆*s*p.* Two constructs were developed, the first under regulation of wildtype promoter (wp) and the second under control of the strong *cytA-p*/STAB-SD promoter system. Lollipop indicates the putative transcriptional terminator site. Oligonucleotide sequences used to delete the signal peptide-coding sequence are shown in Table 1. **(B)** Evaluation of endochitinase activity using solubilized intracellular proteins produced in *E. coli.* Panel 1: Zymogram using 4-MU-(GlcNAc)_3_ for detection. Lane 1, *E. coli-*pEHchiA74∆s*p*; lane 2, without sample; lane 3, *E. coli-*pEBchiA74∆s*p;* lane 4, *E. coli* DH5α*.* Black arrow indicates the position of ChiA74∆s*p* in recombinant *E. coli* strains*.* Panel 2: (a) *E. coli-*pEHchiA74∆s*p*, (b) *E. coli-*pEBchiA74∆s*p.* No endochitinase activity was observed with *E. coli* DH5α*.***(C)** Phase contrast microscopy of recombinant strains of *B. thuringiensis*. Panel 1, 4Q7; panel 2, 4Q7*-*pEHchiA74∆s*p*; panel 3, 4Q7*-*pEBchiA74∆s*p*. Black arrows indicate the positions of ChiA74∆s*p* inclusions. **(D)** Endochitinase activity determined using solubilized intracellular proteins of recombinant strains of *B. thuringiensis.* Panel 1: lane 1, 4Q7; lane 2, 4Q7*-*pEHchiA74∆s*p*; lane 3, 4Q7*-*pEBchiA74∆s*p.* Black arrow indicates the position of ChiA74∆s*p* in recombinant 4Q7 strains*.* Panel 2: (a) 4Q7*-*pEHchiA74∆s*p*, (b) 4Q7*-*pEBchiA74∆s*p*. Activity of recombinant bacteria was normalized with the residual intracellular endochitinase activity of 4Q7.

**Figure 2:**
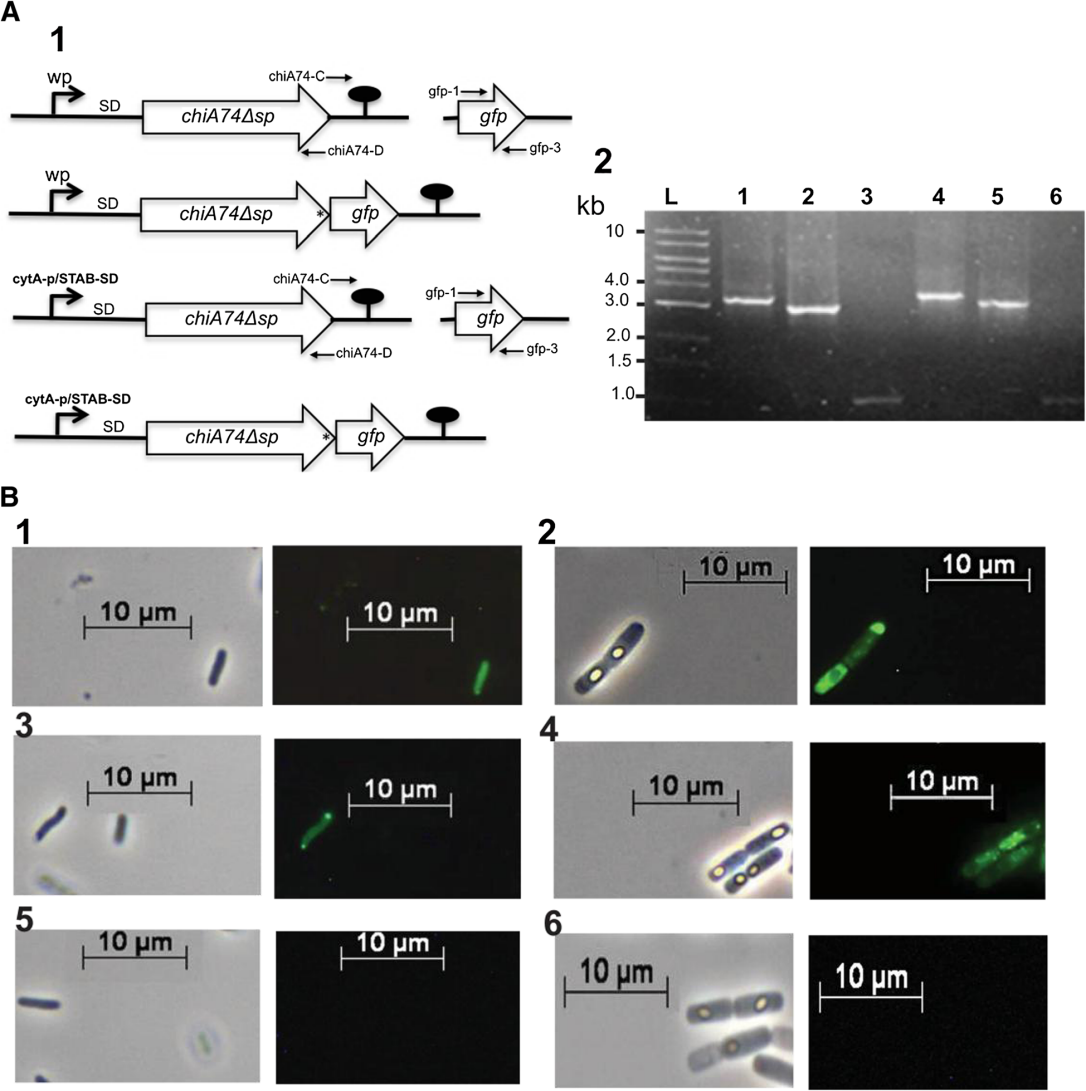
**Confirmation of the intracellular location of ChiA74∆s*p *in *Escherichia coli *and *Bacillus thuringiensis *4Q7 using a chimeric construct with the green fluorescent protein (GFP) gene. (A)** Schematic illustration that shows the fusion of *chiA74*∆s*p* with *gfp*. Panel 1, two constructs were developed, under regulation of the *chiA74* wildtype promoter (wp) or under control of the *cytA*-*p/*STAB-SD system. Lollipop indicates transcriptional terminator. Oligonucleotides used to make chimeric construct with *gfp* are shown in Table 1. Panel 2, confirmation of chimeric constructs by PCR, primers used for amplification are showed in parenthesis and in Table 1. L, 1 kb (kilobase) DNA Ladder (New England Biolabs); Lane 1, pEHchiA74∆*sp*-*gfp* (primers: chiA74-1, chiA74-3); lane 2, pEHchiA74∆*sp*-*gfp* (primers: chiA74-1, gfp-3); lane 3, pEHchiA74∆*sp*-*gfp* (primers: gfp-1, chiA74-4); lane 4, pEB*chi*A74∆sp-*gfp* (primers: cytSTAB-1, chiA74-4); Lane 5, pEB*chi*A74∆sp-*gfp* (primers: cytSTAB-1, gfp-3); lane 6, pEB*chi*A74∆sp-*gfp* (primers: gfp-1, chiA74-4). **(B)** Phase contrast (left) and fluorescence micrographs (right) of recombinant strains of *E. coli* and *B. thuringiensis* strain 4Q7 expressing the chimeric protein ChiA74∆sp-GFP. Panel 1, *E. coli-*pEHchiA74∆*sp*-*gfp*; panel 2, 4Q7*-*pEHchiA74∆*sp*-*gfp*; panel 3, *E. coli-*pEBchiA74∆*sp*-*gfp*; panel 4, 4Q7*-*pEBchiA74∆*sp*-*gfp*; panel 5, *E. coli* DH5α; panel 6, 4Q7. Samples of recombinant strains of *B. thuringiensis* were collected at ~ 72 h.

When *E. coli* was transformed with the constructs lacking the secretion peptide signal sequence coding for amino acids 1–34, i.e. pEHchiA74∆s*p* (native promoter) and pEBchiA74∆s*p* (sporulation-dependent *cytA-p*/STAB-SD promoter) (Figure 1A), recombinant *E. coli-*pEHchiA74∆s*p* and *E. coli-*pEBchiA74∆s*p* showed activities of 150 and 250 mU/mL, respectively; no activity was observed in the control *E. coli* strain (Figure 1B, panel 2). In *in situ* assays using the 4-MU-(GlcNAc)_3_ substrate with proteins fractionated by SDS-PAGE, ChiA74Δsp was detected by zymograms as a protein of *~*70 kDa produced by both recombinant strains (Figure 1B, panel 1), but not in the control strains. To visualize the location of the chitinase in *E. coli*, ChiA74Δsp was fused to the green fluorescent protein (ChiA74Δsp-GFP)*.* Fluorescence was observed within the cytoplasm of the cell, confirming the cytoplasmic location of ChiA74Δsp, the fluorescent chimera frequently accumulated as inclusion bodies at the poles of the cells, as revealed by microscopy (Figure 2B, panels 1, 3, 5). A similar phenomenon has been observed in aging cultures of *E. coli* expressing other heterologous proteins lacking their native secretion signal peptide sequence [18].

### ChiA74Δsp accumulates as intracellular inclusions in acrystalliferous *Bacillus thuringiensis* 4Q7

**Figure 3:**
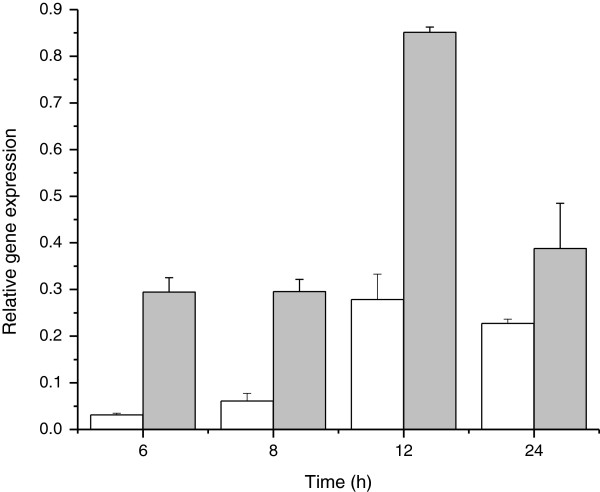
**Relative expression level of *chiA74*Δ*sp *transcripts in 4Q7 at different times.** Expression of the endochitinase gene was regulated by its native promoter (white rectangles) or the strong chimeric sporulation-dependent *cytA-p*/STAB-SD promoters (gray rectangles).

When pEHchiA74Δsp and pEBchiA74Δ*sp* were introduced in 4Q7, the recombinant bacterium produced inclusion bodies in the cytoplasm, readily detected by phase contrast and fluorescence microscopy (Figure 1C; Figure 2B, panels 2, 4). Interestingly, small inclusion bodies dispersed along the cytoplasm appeared to be concentrated at the cell pole before lysis, and were observed as dark bodies by phase contrast microscopy (Figure 1C, panels 2, 3). This phenomenon was confirmed when ChiA74Δsp-GFP was expressed in 4Q7 (Figure 2B, panel 2). When recombinant *B. thuringiensis* 4Q7 cells were disrupted and the intracellular proteins dissolved under alkaline conditions and assayed with the chitin-derived fluorogenic substrate, data normalized with the residual activity in 4Q7 showed that 4Q7*-*pEBchiA74∆sp had an activity of ~ 127 mU/mL, whereas 4Q7*-*pEHchiA74∆sp had and activity of ~ 41 mU/mL. (Figure 1D, panel 2). The higher production of ChiA74∆sp in 4Q7*-*pEBchiA74∆sp could be attributed to the endochitinase gene expressed using the strong *cytA-p/STAB* promoter as quantitative PCR showed the relative amount of *chiA74∆sp*-specific mRNA increased by ~9-, 5-, 3- and 2-fold when compared to expression with the native promoter at 6, 8, 12 and 24 h, respectively. We note that the highest mRNA *chiA74*Δ*sp* expression was observed at 12 h (Figure 3). In addition, ChiA74∆sp was detected by zymograms in both recombinant strains as a protein of ~70 kDa. Under the time of UV exposure, we did not observe a fluorescence signal produced by native 4Q7 chitinases (Figure 1D, panel 1).

### ChiA74Δsp forms stable inclusions in HD1

**Figure 4:**
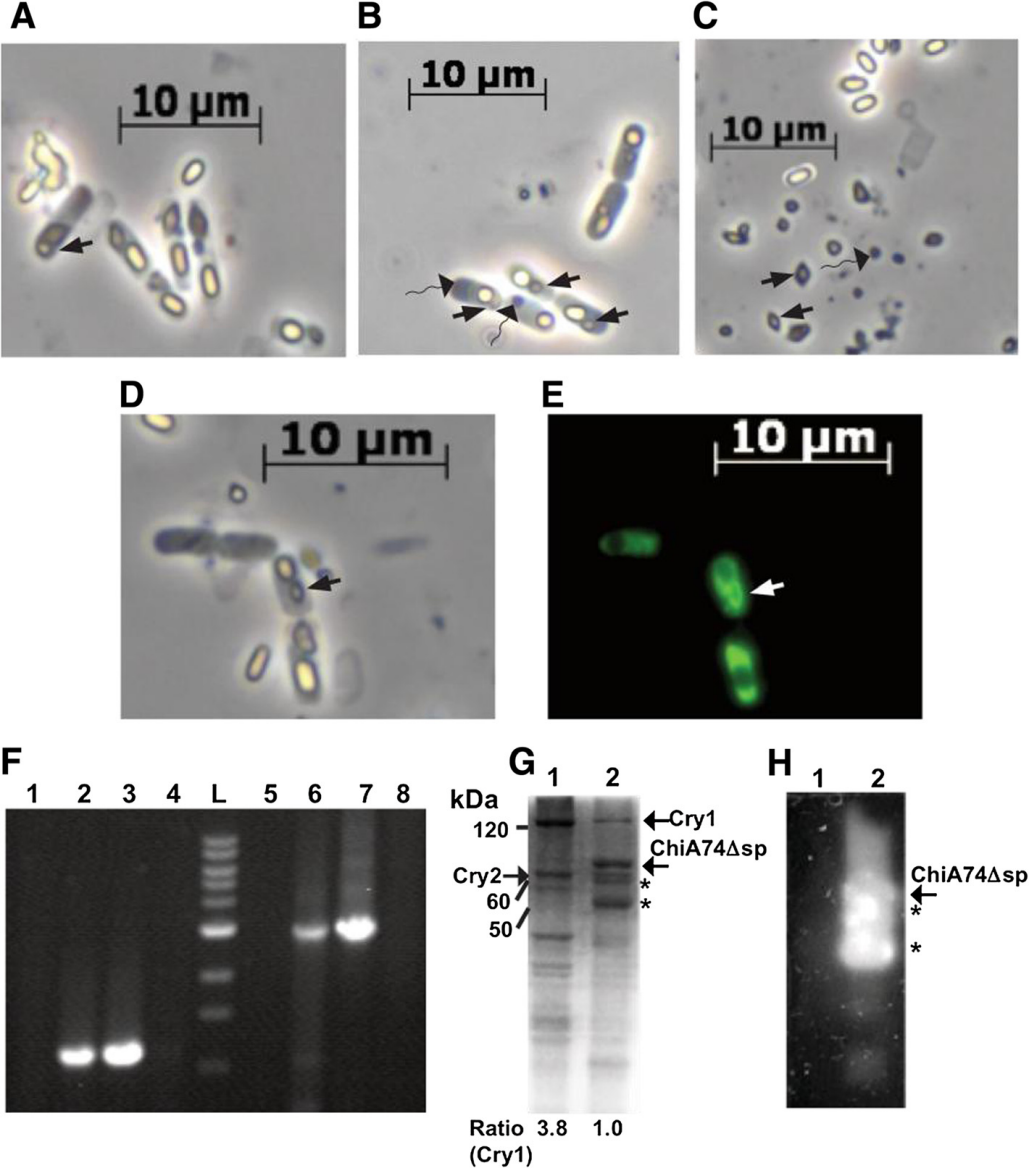
**Expression of ChiA74Δsp in *B. thuringiensis *HD1. (A)** Phase contrast micrograph of HD1 sporangia, **(B)** HD1-pEBchiA74∆sp sporangia, **(C)** HD1-pEBchiA74∆sp lysed culture. Triangle and triangle-wide wavy arrows indicate the presence of Cry crystals and ChiA74∆sp inclusion bodies, respectively. In **(A)** arrow shows a bipyramidal crystal (Cry1) associated with a cuboidal crystal (Cry2). ChiA74∆sp was fused to the GFP and expressed in HD1 to obtain HD1-pEBchiA74*∆sp*-gfp; **(D)** Phase contrast, and **(E)** fluorescence microscopy, black and white arrows indicate the crystal position. Fluorescence along the sporangium indicates that ChiA74∆sp was expressed and accumulated within the cell. **(F)** Confirmation of the transformation of HD1 by PCR. Lanes 1 and 5, HD1; Lanes 2 and 6, HD1-pEBchiA74*∆sp*; Lanes 3 and 7, *E. coli*-pEBchiA74*Δsp*; lanes 4 and 8, pBluescript KS (+) (Stratagene); L, 1 kb DNA Ladder (10, 8, 6, 5, 4, 3, 2, 1.5, 1 kb; New England Biolabs). Amplicons of lanes 1 to 4 and 5 to 8 were obtained using the primers ery1, ery2, and cytSTAB-1, chiA74-4, respectively. **(G)** SDS-PAGE and **(H)** zymogram of solubilized proteins obtained from sporulated and lysed HD1 (lane 1) and HD1-pEBchiA74*∆sp* (lane 2). Location of Cry1A, Cry2A proteins and endochitinase (ChiA74∆sp) are showed with black arrows. A reduction in the relative amount of Cry1 (~133 kDa) and Cry2A (~65 kDa) proteins was observed in the recombinant strain (lane 2) compared with wildtype HD1 (lane1). Zymogram detection was performed using the 4-MU-(GlcNAc)_3_ substrate. Asterisks indicate probable ChiA74∆sp degradation products. Protein molecular masses were deduced using the reference BenchMark protein ladder standard (Invitrogen, Carlsbad CA, USA).

The initial studies in *E. coli* and 4Q7 demonstrated that ChiA74Δsp could be stably produced as intracellular inclusions in these bacteria. To further our studies, we transformed HD1 with pEBchiA74∆s*p* and confirmed the fidelity of the recombinant by PCR using specific oligonucleotides (Table 1) to detect the erythromycin resistance gene (~1 kb) and the endochitinase gene under control of the *cytA-p/STAB-SD* system (~3 kb); both genes were not detected in wildtype HD1 or pBluescript KS(+) (Stratagene) which were used as negative controls (Figure 4F). When HD1-pEBchiA74*Δsp* was observed by phase contrast microscopy, ChiA74Δsp inclusion bodies, most commonly occurring at the sporangium pole, could be easily distinguished from the native bipyramidal crystals and endospores (Figure 4A,B,C). Fluorescence microscopy of ChiA74*Δsp-*GFP confirmed the intracellular location of the inclusion (Figure 4D,E).

**Table 1:** **Primers used for PCR construction and amplification of *chiA74*∆s*p *** **and *chiA74*∆s*p-gfp***

**Primer**	**Sequence**^**a**^
chiA74-13	F: 5′-TCC*CCGCGG***ATG**TCACCAAAGCAAAGTCAAAAAATTGTTGGGTAC-3′
chiA74-12	R: 5′-TCC*CCGCGG*TTCTCCTTTCAAAATAAAAGATATATTTAAAGGC-3′
gfp-1	F: 5′- ATGGCTAGCAAAGGAGAAGAACTTT-3′
gfp-3	R: 5′- GGTC*AGATCT*TTATTTGTAGAGCTCATCCAT -3′
chiA74-C	F: 5′- GGTC*AGATCT*ACGTAATATCCATTAATTACTTCACTA -3′
chiA74-B	R: 5′- GTTTTCGCTAATGACGGCATTTAAAAG -3′
cyt-STAB-1	F: 5′-CGGAATTCTATTTTCGATTTC-3′
chiA74-3	R: 5-AA*CTGCAG*CGAAAGCCTTTCCCTAACAGGTGACTATC-3′
ery-1	F: 5-AAAA*CTGCAG*CTTAAGAGTGTGTTGATAGTGC-3′
ery-2	R: 5-ATAAGAAT*GCGGCCGC*CCCCGTAGGCGCTAGGGACC-3

^a^Artificial start ATG codon (bold type) used to initiate the chitinase translation, and the restriction endonuclease cleavage sites SacII, BglII, PstI and NotI (italics) are shown.

**Figure 5:**
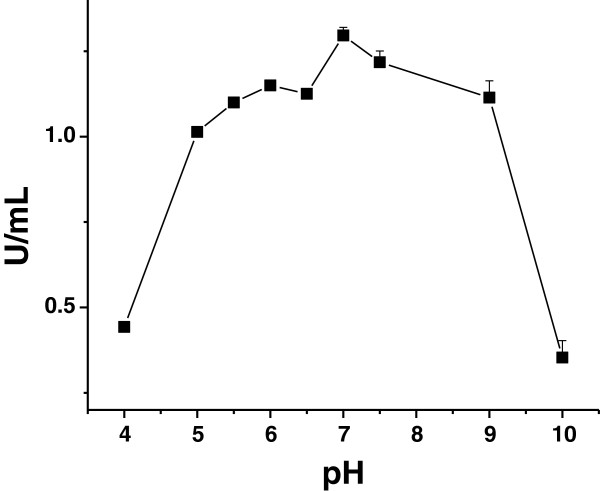
**Effect of pH on endochitinase activity of alkaline-solubilized ChiA74Δsp inclusion bodies produced in recombinant *B*. *thuringiensis *HD1.** Lysed culture (~74 mL) of recombinant bacteria were solubilized in an alkaline and reducing solution and then evaluated against 4-MU-(GlcNAc)_3_ at a pH range of 4–10.

The effect of ChiA74*Δsp* synthesis on Cry crystal size in HD1-pEBchiA74*Δsp* was also determined. A reduction by ~33% in area of the Cry crystalline inclusion was observed in the recombinant producing ChiA74*Δsp* when compared to crystals produced by wildtype HD1 (Table 2). Although the area (two-dimensional surface) determination is not suggestive of volume (three-dimensional space) to determine the yield of Cry proteins, the decrease in the crystal area correlated well with the reduction in the relative amount of Cry1 (~133 kDa)/Cry2Aa (~65 kDa) proteins detected by SDS-PAGE (Figure 4G). In addition, a band corresponding to endochitinase ChiA74Δsp (~70 kDa), and other smaller bands which could correspond to endochitinase degradation, were observed in the recombinant but not in HD1, as confirmed by zymogram analyses (Figure 4H). The chitinase activity was markedly increased (~42-fold) in HD1-pEBchiA74*Δsp* when compared with HD1, respectively, ~127 mU/mL and 3 mU/mL (Table 2). Moreover, we determined the activity of the recombinant chitinase in a range of pH typically observed in lepidopteran larval midgut (~ pH 8–11). The enzyme retained its activity at a range from pH 5 to 9, but it was reduced drastically to ~70% at pH 10 (Figure 5). We also note that at the same period of growth (72 h) in nutrient broth, the viable spore count for the recombinant was ~3-fold greater when compared with HD1 (Table 2).

**Table 2:** Endochitinase activity (U)*, crystal area and viable spores of wildtype and recombinant strains of *Bacillus thuringiensis*

	**HD1 *-*pEBchiA74∆sp**	**HD1**
mU/mL (±SD)	127 (± 2.0)^a**^	3.0 (± 0.2)^b^
Ratio	42.0	1.0
Crystal area (mμ^2^) (±SD)	0.86 (± 0.16)^a^	1.28 (± 0.19)^b^
Ratio	1.0	1.5
Spores/mL x 10^7^ (±SD)	8.35 (± 0.57)^a^	2.95 (± 0.21)^b^
Ratio	2.83	1.0

*One unit (U) was defined as the amount of enzyme required to release 1 μmol of 4-methylumbelliferone in 1 h.

**Values with different letter (^a, b^) in the same row are significantly different as determined by Tukey’s multiple range test (*P* < 0.05).

### Bioassays

Spore-Cry crystal mixtures of HD-1 and spore-Cry crystal-ChiA74Δsp inclusion mixtures of HD1*-*pEBchiA74∆sp were assayed against first instar larvae of the tobacco hornworm (*Manduca sexta*). The LC_50_ for HD1 was 67.30 ng/cm^2^ diet and 41.45 ng/cm^2^ diet for HD1*-*pEBchiA74∆sp, representing an apparent 1.6x enhancement in toxicity of the recombinant strain. However, we did not detect a significant difference in the LC_50_s as there was an overlap between the upper fiducial limit of the recombinant strain’s LC_50_ and the lower fiducial limit of the wildtype LC_50_. A difference of 25.84 ng/cm^2^ diet between both LC_50s_, only showed that the recombinant strain required lower concentration than the wildtype to kill 50% of the larvae (Table 3). In addition, no toxicity was observed (0% mortality) against *M. sexta* using spores-ChiA74Δsp inclusion mixtures synthesized in 4Q7 (data not shown).

**Table 3:** **Statistical parameters for estimating the LC**_**50 **_**of strains of *B. thuringiensis *** **against tobacco hornworm *Manduca sexta***

**Strain**	**LC**_**50**_^**a**^	**Slope**	**X**^**2**^	**Mortality**
HD1	67.29 (47.18-95.98)	1.70	0.90	0
HD1-pEBchiA74∆sp	41.45 (28.33-60.64)	1.50	0.17	0

^a^Values are shown in nanograms per cm^2^ of spore-crystal mixture or spore-crystal-ChiA74∆sp inclusion bodies mixture for HD1 and HD1-pEBchiA74∆sp, respectively, and represent 5-days mortality as determined by Probit analysis. Fiducial limits are indicated in parenthesis.

## Discussion

The use of bacterial chitinases could be of significance in *Bacillus thuringiensis*-based biocontrol efforts because they synergize insecticidal Cry proteins produced by strains of this species [19-23]. Although increases in synthesis of extracellular chitinases in *B. thuringiensis* has been accomplished using various expression systems*,* the practical problem regarding the likely instability of potential mixtures of spore-crystal-soluble chitinase formulations remains to be resolved [9,16]. Ideally, the production of physically stable, but biochemically (alkaline) labile, inclusions of chitinase and Cry crystals in the same cell could alleviate this concern. Hu *et al*. [11] successfully produced chitinase lacking its secretion signal peptide sequence as inclusions in an acrystalliferous *B. thuringiensis* strain. However, the concern was not resolved as chitinase inclusions and Cry crystals were synthesized in different bacterial strains of which preparations must be mixed for bioassays, or for prospective commercial formulations. In addition, their work as designed could not address the effect of chitinase synthesis on Cry crystal production and viable spore count of recombinant *B. thuringiensis*, two factors that must be optimized for potential applied and commercial consideration.

In the present study, we demonstrated that by deleting the secretion signal peptide sequence of ChiA74 (ChiA74∆sp)*,* stable occluded ChiA74∆sp could be produced in different bacteria. First, we transformed *E. coli* with the constructs to produce sufficient recombinant plasmid DNA to transform *B. thuringiensis.* To our surprise we observed the formation of small inclusion bodies at the poles of *E. coli* and demonstrated they were composed of ChiA74∆sp. To our knowledge it is the first report that chitinase inclusion bodies can be produced in *E. coli.* We note that the synthesis of ChiA74∆sp as stable inclusions in *E. coli* could have biotechnological value, as it could be mass-produced and easily purified using an organism “generally recognized as safe” (e.g. *E. coli* K12) for applied used, such as for generating chitin-derived oligosaccharides with pharmaceutical and/or food preservation properties [10].

Our major objective in this work was to produce, for first time, ChiA74∆sp inclusions together with insecticidal crystals and spores in the same cell, study its cellular effect and determine the recombinant’s toxicity to an important agronomical insect such as *M. sexta* larvae. We observed the formation of ChiA74∆sp inclusions in the acrystalliferous *B. thuringiensis* 4Q7, and like in *E. coli,* they accumulated at the poles. Increased chitinase synthesis was observed when the endochitinase gene was expressed using the strong *cytA-p/STAB* promoter system developed by Park et al. (1998) (17), compared to regulation by its native promoter, and most likely was a result of increased *chiA74*∆sp mRNAs, as demonstrated by qPCR. Interestingly, when we transformed the crystalliferous strain *B. thuringiensis* HD1 with the endochitinase gene *chiA74*∆sp regulated by *cytA-p/STAB*, we observed two important changes in the recombinant strain: (i) a reduction in the crystal size (i.e. less Cry production) and (ii) a 3-fold increase in the number of viable spores*.* With regards to crystals, we observed an ~33% decrease in the Cry crystals area (Figure 4A,B,C; Table 2) similar to previous reports [9,16], which correlated well with a decrease in Cry protein synthesis as detected by SDS-PAGE (Figure 4G). The increase in spore count was not expected based on results of a previous study where the opposite occurred following expression of heterologous chitinase (secreted) in *B. thuringiensis*[16]. Although we do not have supporting experimental evidence, it is possible that the synthesis of two kind of proteins, Cry and ChiA74, whose gene expression is controlled by two strong sporulation-dependent promoters (BtI-BtII, *cyt-p/STAB*) incur a more rapid depletion of nutrients thereby inducing sporulation. For example, it is known that the activation of Spo0A, a master regulator for entry into sporulation in *B. subtilis,* is induced in response to nutrient limitation [24,25].

As suggested previously, the advantage of producing ChiA74∆sp inclusions in HD1 allow the direct use of spore-Cry crystals-ChiA74∆sp mixtures from a single source in bioassays, rather than mixing preparations from different strains as reported previously [11]. We were successful in engineering a recombinant HD1 strain producing ChiA74∆sp inclusions during sporulation that had a 42-fold increase in chitinase activity. Despite the marked increase in chitinase activity, only an apparent 1.6-fold increase in toxicity was observed against *M. sexta* first instar larvae. Similar results (1.5-fold increase) have been observed with mixtures of different recombinant strains producing chitinase inclusions and Cry1Ac against *S. exigua* and *H. armigera*[11]. It is likely that the decrease in Cry crystal synthesis in the recombinant strain lowered the expectations of several-fold increases in insecticidal activity of the recombinant. In addition, and probably of more significance, is the marked decrease (~70%) in enzymatic activity of preparations of ChiA74Δsp inclusions solubilized at pH 10 (Figure 5). It is worth noting that lepidopteran midguts normally show pH gradients from anterior to posterior, and from the lumen to epithelial microvilli. The midgut of *M. sexta* larva ranges in pH from ~10-11 [26]. Although it is evident that the lower LC_50_ of the recombinant strain is a consequence of an increase chitinase production with the compensating decrease in Cry crystal proteins, our results suggest that more “balanced” expression of both *cry* and *chiA74Δsp* could result in optimal production of these proteins conducive to an efficacious biopesticide.

In summary, we have produced ChiA74∆sp inclusion in HD1 and the recombinant showed an apparent increased activity against first instar *M. sexta* larvae. Our future studies include producing ChiA74∆sp inclusions in other lepidopteran-, coleopteran- and dipteran-specific strains of *B. thuringiensis* for bioassays against a wide variety of insect larvae. Finally, we are also in the process of using molecular strategies to modify expression of ChiA74∆sp, while at the same time maximizing the production of endogenous Cry proteins to develop highly efficacious strains of *B. thuringiensis* for applied use.

## Conclusions

Inclusions of ChiA74Δsp can be produced in *E. coli* and *B. thuringiensis* strains. We show for the first time, the ability to synthesize ChiA74∆sp inclusions, insecticidal Cry crystals and spores in the same sporangium. We observed that the production of ChiA74∆sp inclusions affect the crystal size and sporulation in *B. thuringiensis* subsp. *kurstaki* HD1. The data reported in this study lay a foundation for developing strategies to modify expression of *chiA74∆sp* while maximizing the production of Cry proteins.

## Material and methods

### Bacterial strains and plasmids

Plasmids pEHchiA74 and pEBchiA74 harbor the *chiA74* under the control of, respectively, the wild promoter (wp) and the 660-bp strong chimeric sporulation-dependent *pcytA-p*/STAB-SD promoter developed by Park et al. (1998) [17]. The wildtype Shine-Dalgarno (wSD) and transcription terminator (*chiA74tt)* sequences were retained in all constructs [9,16]. These plasmids (pEHchiA74 and pEBchiA74) were used for deleting the signal peptide-encoding sequence of ChiA74 to obtain ChiA74Δsp (see below). All constructs (Figure 1A) were propagated in *E. coli* DH5α [*supE44, DlacU169* (F80*lacZDM15*) *hsdR17 recA1 end A1 gyrA96 thi-1 relA1*] (Invitrogen, Carlsbad CA, USA) and then used for transforming the acrystalliferous strain of *B. thuringiensis* subsp. *israelensis* 4Q7, (hereafter 4Q7), and *B. thuringiensis* subsp. *kurstaki* HD1 (hereafter HD1) (*Bacillus* Genetic Stock Center, Columbus, OH)*.* Plasmid pGLO is a vector that harbors the green fluorescent protein (*gfp*) gene under the control arabinose (*araC*) promoter and contains an ampicillin (*bla*) resistance gene marker (Bio-Rad, Hercules CA, USA). The shuttle vectors used to transform the different constructs in *B. thuringiensis* were the pHT3101 and the pSTAB, a pHT3101-derived vector containing *cyt1A-*p/STAB-SD (17), both harbor erythromycin and ampicillin resistance gene markers [27].

### Construction of recombinant plasmids

#### ChiA74 without the signal peptide sequence under control of the wildtype promoter (chiA74Δsp-wp) or the cyt1A-p/STAB-SD system (chiA74Δsp-cyt1A-p/STAB-SD)

Recombinant plasmid pEHchiA74 and pEBchiA74 harboring the *chiA74* under the control of the wild promoter and the *pcytA-p/*STAB-SD, were used as templates, respectively. Two primers (chiA74-13 F and chiA74-12R) were designed to amplify *chiA74* without the signal peptide-encoding sequence (i.e., codons 1–34 deleted). ChiA74-13 F contains an artificial *Sac*II site at the 5′ end, an artificial ATG translation initiation codon in frame with the remaining sequence of *chiA74* (starting with the codon for Ser-35). ChiA74-12 F contains an artificial *Sac*I site in the 5′ of the lower strand and was used to amplify *chiA74* (Figure 1A, Table 1). PCR amplification was performed with the Phusion High-Fidelity DNA Polymerase (Finnzymes, Finland) in a C1000 Touch Thermal Cycler (Bio-Rad, Hercules, CA, USA). Amplicons were purified using the QIAquick gel extraction kit (Qiagen, Valencia, CA, USA), treated with T4 polynucleotide kinase (New England BioLabs, Beverly, MA) and then ligated with T4 DNA ligase (New England BioLabs, Beverly, MA). Deletion of the signal peptide-encoding sequence was confirmed by PCR and nucleotide sequencing of the recombinant plasmids. Recombinant plasmids harboring the *chiA74Δsp*-*wp* or the *chiA74Δsp*-*cyt1A-*p/STAB-SD were designated as pEHchiA74*Δsp* and pEBchiA74*Δsp*, respectively.

#### chiA74Δsp-GFP fusion

The open reading frame coding for the green fluorescent protein (*gfp*) was amplified from the pGLO vector using the gfp-1 and gfp-3 primers (see list of primers in Table 1). PCR amplification was performed with the Phusion High-Fidelity DNA Polymerase (Finnzymes, Finland). In addition, *chiA74Δsp*, lacking the stop codon, under the control of the wild or *cytA-p* promoters was amplified from pEHchiA74*Δsp* and pEBchiA74*Δsp*, respectively, using the primers chiA74-C (forward) and chiA74-B (reverse). Then the amplicons pEHchiA74Δ*sp*, pEBchiA74Δ*sp* and *gfp* were digested with *Bgl*II*,* ligated and then used to transform *E. coli* DH5α to obtain the chimeric constructs pEHchiA74Δ*sp*-*gfp* and pEBchiA74Δ*sp-gfp* (Figure 2A1). The fidelity of constructs was confirmed by restriction enzyme and PCR analyses using specific primers (Figure 2A2).

### Transformation

Recombinant plasmids were introduced into *E. coli* DH5α using an *E. coli* pulser (BioRad) set at 2.5 kV, 200 Ω and 25 μF and transformants were selected on Luria-Bertani (LB) medium with ampicillin (100 μg mL^-1^) (10). 4Q7 and HD1 competent cells were prepared as described previously [28]. Approximately 3 μg of the recombinant plasmids were mixed with 300 μl of competent cell suspension, held on ice for 10 min followed by electroporation using a BTX ECM630 electro cell manipulator (San Diego CA, USA) set at 2.3 kV, 475 Ω and 25 μF. After the pulse, the suspension was added to 3 ml of brain heart infusion (BHI) (Bioxon México) and incubated with gentle shaking for 1 h at 37°C. Transformants were selected on BHI supplemented with 25 μg mL^-1^ of erythromycin.

### Phase contrast and fluorescence microscopy

*E. coli*, 4Q7 and HD1 were cultivated in LB or nutrient broth at 37°C or 28°C (200 rpm), respectively. Samples were taken at different times and monitored by phase contrast and fluorescence microscopy. Data were obtained using an Axio Imager A.1 Zeiss microscope with the filter set at 09, an excitation of 450–490 nm, and an emission of 515 nm. Crystal area was estimated using the AxioVision LE program (Carl Zeiss Microscopy, Göttingen Germany).

### Evaluation of the chitinase activity

To determine the level of endochitinase activity in preparations of *E. coli-*pEHchiA74∆sp, *E. coli-*pEBchiA74∆sp and 4Q7-pEBchiA74Δ*sp*, 4Q7-pEBchiA74Δ*sp*, bacteria were cultivated in LB with ampicillin (100 μg/ml) at 37°C, 200 rpm, or in nutrient broth with erythromycin (25 μg/ml) at 28°C, 200 rpm [16], respectively. Controls (*E. coli* and 4Q7) were grown without antibiotics. Cultures were centrifuged, washed three times with distilled water and resuspended in 100 mM phosphate buffer (pH 7.0). Cells were sonicated three times, 15 s each, at an amplitude of 40 Hz in a 20 kHz ultrasonic processor (Sonics and Materials, Inc). Samples were centrifuged and the pellets mixed with solubilization buffer (30 mM Na_2_CO_3_, 0.2% β-ME, 1 mM phenylmethylsulfonyl fluoride, pH 10–11 [11]. Suspensions were incubated at 37°C with gentle agitation for 40 min, centrifuged, and supernatants were assayed with the fluorogenic substrate 4-MU-(GlcNAc)_3_ at pH 6.8, in a Glomax Multi Jr. Detection System (Promega, Sunnyvale CA, USA), as previously described [29]. To determine chitinase activity of HD1 and HD1-pEBchiA74Δ*sp*, bacteria were growth in 75 mL nutrient broth with or without erythromycin, respectively, and incubated at 28°C, 200 rpm to autolysis (~72 hr). To compare activity of the recombinant *versus* wildtype strain, 1 mL of each culture was centrifuged and the pellets were washed three times with distilled water and then resuspended in 150 μL of solubilization buffer. Samples were incubated at 37°C with gentle agitation for 40 min, centrifuged, and supernatants were assayed with the fluorogenic substrate 4-MU-(GlcNAc)_3_ at pH 6.8. In addition, activity at different pH of the alkaline-solubilized recombinant chitinase was determined. Approximately 74 mL of the remaining culture was centrifuged, washed with distilled water and resuspended in 5 mL of solubilization buffer. The enzymatic activity of concentrated ChiA74Δsp at a pH range of 4–10 was evaluated with the tetrameric fluorogenic derivative using a reaction buffer containing sodium acetate, MES [2 (N-morpholino) ethane sulfonic acid], NaH_2_PO_4_, Trizma base [Tris(hydroxymethyl) aminomethane] and glycine, with a final concentration of 15 mM for each component.

In addition, dissolved ChiA74Δsp samples were fractionated in a 12% polyacrylamide gel by sodium dodecyl sulfate-polyacrylamide gel electrophoresis (SDS-PAGE). Afterwards proteins were renatured by removing SDS and 2-mercaptoethanol with casein-EDTA wash buffer (1% casein, 2 mM EDTA, 40 mM Tris–HCl, pH 9). Detection of chitinase activity was determined using 4-MU-(GlcNAc)_3_, as previously described [29].

### Effect on viable spores count

Bacteria were growth in nutrient broth for 3 days at 28°C, 200 rpm. Then 100 μL of autolysed cultures were incubated at 60°C for 20 min to destroy remaining vegetative cells [16]. After serial dilution (10^-5^-10^-6^), suspensions were plated on nutrient agar with or without erythromycin and incubated at 28°C for 24 h to determine the number of viable spores. Data were analyzed with the ANOVA program (StatSoft Inc.).

### Quantitative PCR (qPCR)

Total mRNA was obtained from 4Q7-pEHchiA74Δ*sp*-*gfp* and 4Q7-pEBchiA74Δ*sp-gfp.* One mL of each bacterial culture was harvested periodically from 2 h to 96 h, centrifuged and cells were resuspended in 1 mL of Trizol (Invitrogen, Carlsbad CA, USA). Samples were sonicated 15 s in an ultrasonic processor (Sonics and Materials, Inc), and RNA extraction was performed according to manufacturer’s protocol (Invitrogen, Carlsbad CA, USA). Total RNA was resuspended in 30 μL of double distilled water and DNA contamination was eliminated using DNAse I (Jena Bioscience, Jena Germany). Then 1 μg of total RNA was used to synthesize cDNA using the iScript cDNA synthesis kit according to the manufacturer’s instruction (Bio-Rad, Hercules CA, USA). For quantitative PCR, specific primers were used to amplify the erythromycin and green fluorescent protein gene (*gfp*). The erythromycin gene was used as internal control to normalize the RNA. As the chitinase gene in 4Q7 is amplified with the specific primer of *chiA74*Δ*sp* (data not shown), the *gfp* was employed to determine the relative amount of *chiA74*Δ*sp* mRNA in the recombinant bacteria because this gene was fused to the *chiA74*Δ*sp*. Quantitative PCR was carried out in the CFX connect Real time system (Bio-Rad, Hercules CA, USA). Reaction mixture contained 5 μL of SyBR green master mix, 0.4 mM of each primer and 40 μg/mL of total transcribed RNA. Thermal cycling conditions were: 95°C for 5 min, followed by 40 cycles of 95°C for 30 s and 55°C for 60 s. This was followed by a melting curve program of 65 to 95°C with a heating rate of 0.5°C per second. Data were analyzed by relative quantification using the ΔC_T_ method (Bio-Rad, Hercules CA, USA).

### Bioassays

*Manduca sexta* (Lepidotpera: *Sphingidae*) colonies were maintained on artificial diet [30] under laboratory conditions at 28 ± 2°C and 70 ± 10% relative humidity, under a 16:8 (light:dark) photoperiod. Strains were cultured in nutrient broth at 28°C, 200 rpm. Then sporulated and autolyzed cultures were centrifuged and supernatants were discarded to eliminate secreted molecules such as protease, endogenous chitinases and putative Vip proteins. Pellets (spore-Cry crystal mixtures of HD-1 and spore-Cry crystal-ChiA74Δsp inclusion mixtures of HD1*-*pEBchiA74∆sp) were washed three times with distilled water, lyophilized and powders were used for bioassays. Six different preparations of HD1 and HD1-pEBchiA74∆sp, and a tap water negative control, were assayed in triplicate. A constant volume of the sample dilution (250 μl) was applied onto the surface of diet contained in Petri dishes (area 60 cm^2^). Ten first instar larvae were added to each Petri dish and mortality was recorded after five days of incubation under laboratory conditions. The mean concentration at which 50% (LC_50_) of the larvae died was estimated by Probit analysis [31].

## Competing interests

The authors declare that they have no competing interests.

## Authors’ contributions

JEBC designed the experimental setup, obtained financial support, initiated the project, analyzed results and wrote the manuscript. JEBC, JLDA, JCCR, UEBP, LECV and MCRC performed the different experiments. DKB and MCRC help to design the experimental setup and in manuscript preparation. All authors read and approved the final manuscript.

## References

[B1] Park H-W Federici BA Sakano Y Shively JM Insecticidal protein crystals of *Bacillus thuringiensis* Microbiology monographs: inclusions in prokaryotes, Volume 1 2006 Heidelberg, Germany: Springer-Verlag 195 236

[B2] Wu D Federici BA A 20-kilodalton protein preserves cell viability and promotes CytA crystal formation during sporulation in *Bacillus thuringiensis* J Bacteriol 1993 175 5276 5280 8349568 10.1128/jb.175.16.5276-5280.1993 PMC204998

[B3] Federici BA Insecticidal bacteria: an overwhelming success for invertebrate pathology J Invertebr Pathol 2003 89 30 38 16039303 10.1016/j.jip.2005.06.007

[B4] Sanahuja G Banakar R Twyman RM Capel T Chrstou P *Bacillus thuringiensis:* a century of research, development and commercial applications Plant Biotechnol J 2011 9 283 300 10.1111/j.1467-7652.2011.00595.x 21375687

[B5] Aronson A Sporulation and *d*-endotoxin synthesis by *Bacillus thuringiensis* Cell Mol Life Sci 2002 59 417 425 10.1007/s00018-002-8434-6 11964120 PMC11337470

[B6] Bietlot HPL Vishmulhatla J Carey PR Pozsgay M Kaplan H Characterization of the cysteine residues and disulphide linkages in the protein crystal of *Bacillus thuringiensis* Biochem J 1999 267 309 315 2110449 10.1042/bj2670309 PMC1131288

[B7] Chan L Grant R Aronson A Regulation of the packaging of *Bacillus thuringiensis* Δ-endotoxins into inclusions Appl Environ Microbiol 2001 67 032 5036 10.1128/AEM.67.11.5032-5036.2001 PMC93267 11679322

[B8] Schnepf E Crickmore N Van Rie J Lereclus D Baum J Feitelson J Zeigler DR Dean DH *Bacillus thuringiensis* and its pesticidal crystals proteins Microbiol Mol Biol Rev 1998 62 775 806 9729609 10.1128/mmbr.62.3.775-806.1998 PMC98934

[B9] Casique-Arroyo G Bideshi D Salcedo-Hernández R Barboza-Corona JE Development of a recombinant strain of Bacillus thuringiensis subsp. kurstaki HD-73 that produces the endochitinase ChiA74 Antonie Van Leeuwenhoek 2007 92 1 9 10.1007/s10482-006-9127-1 17136568

[B10] Castañeda-Ramírez C De la Fuente-Salcido NM Salcedo-Hernández R León-Galván F Bideshi DK Barboza-Corona JE High-level synthesis of endochitinase ChiA74 in *Escherichia coli* K12 and its promising potential for use in biotechnology Folia Microbiologica 2013 58 455 462 10.1007/s12223-013-0229-7 23400505

[B11] Hu SB Liu P Ding XZ Yan L Sun YJ Zhang YM Li WP Xia LQ Efficient constitutive expression of chitinase in the mother cell of *Bacillus thuringiensis* and its potential to enhance the toxicity of Cry1Ac protoxin Appl Microbiol Biotechnol 2009 82 1157 1167 10.1007/s00253-009-1910-2 19277644

[B12] Kuzu SB Güvenmez HK Denizci AA Production of a thermostable and alkaline chitinase by *Bacillus thuringiensis* subsp. *kurstaki* strain HBK-51 Biotechnol Res Int 2012 Article ID 135498. doi:10.1155/2012/135498. 10.1155/2012/135498 PMC3532916 23304523

[B13] Thamthiankul S Moar WJ Miller ME Panbangred W Improving the insecticidal activity of *Bacillus thuringiensis* subsp *aizawai* against *Spodoptera exigua* by chromosomal expression of a chitinase gene Appl Microbiol Biotechnol 2004 65 183 192 15107949 10.1007/s00253-004-1606-6

[B14] Ding X Luo Z Xia L Gao B Sun Y Zhang Y Improving the insecticidal activity by expression of a recombinant *cry1Ac* gene with chitinase-encoding gene in acrystalliferous *Bacillus thuringiensis* Curr Microbiol 2008 56 442 446 10.1007/s00284-008-9112-1 18259812

[B15] Driss F Rouis S Azzouz H Tounsi S Zouari N Jaoua S Integration of a recombinant chitinase into *Bacillus thuringiensis* parasporal insecticidal crystal Curr Microbiol 2011 62 281 288 10.1007/s00284-010-9704-4 20625731

[B16] Barboza-Corona JE Ortiz-Rodríguez T de la Fuente-Salcido N Ibarra J Bideshi DK Salcedo-Hernández R Hyperproduction of chitinase influences crystal toxin synthesis and sporulation of *Bacillus thuringiensis* Antonie Van Leeuwenhoek 2009 96 31 42 10.1007/s10482-009-9332-9 19337851

[B17] Park HW Ge B Bauer LS Federici BA Optimization of Cry3A yields in *Bacillus thuringiensis* by use of sporulation-dependent promoters in combination with the STAB-SD mRNA sequence Appl Environ Microbiol 1998 64 3932 3938 9758822 10.1128/aem.64.10.3932-3938.1998 PMC106581

[B18] Coquel AN Jacob JP Primet M Demarez A Dimiccoli M Julou T Moisan L Lindner AB Berry H Localization of protein aggregation in *Escherichia coli* is governed by diffusion and nucleoid macromolecular crowding effect PLoS Comput Biol 2013 9 4 e1003038 10.1371/journal.pcbi.1003038 23633942 PMC3636022

[B19] Buasri W Panbangred W Large crystal toxin formation in chromosomally engineered *Bacillus thuringiensis* subsp. *aizawai* due to σ^E^ accumulation Appl Environ Microbiol 2012 78 1682 1691 10.1128/AEM.06505-11 22267677 PMC3298149

[B20] Cai Y Yan J Hu X Han B Zhiming Yuan Z Improving the insecticidal activity against resistant *Culex quinquefasciatus* mosquitoes by expression of chitinase gene *chiAC* in *Bacillus sphaericus* Appl Environ Microbiol 2007 73 7744 7746 10.1128/AEM.01510-07 17933917 PMC2168083

[B21] Ozgen A Sezen K Demir I Demirbag Z Nalcacioglu R Molecular characterization of chitinase genes from a local isolate of *Serratia marcescens* and their contribution to the insecticidal activity of *Bacillus thuringiensis* strains Curr Microbiol 2013 67 499 504 23728785 10.1007/s00284-013-0395-5

[B22] Smirnoff WA Effect of chitinase on the action of *Bacillus thuringiensis* Can Entomol 1971 12 1829 1831

[B23] Tang Y Tong J Zhang Y Wang L Hu S Li W Lv W Preliminary comparing the toxicities of the hybrid *cry1Ac*s fused with different heterogenous genes provided guidance for the fusion expression of Cry proteins World J Microbiol Biotechnol 2012 28 397 400 10.1007/s11274-011-0825-0 22806817

[B24] Fujita M González-Pastor JE Losick R High-and low-threshold genes in the Spo0A regulon of *Bacillus subtilis* J Bacteriol 2005 187 1357 1368 10.1128/JB.187.4.1357-1368.2005 15687200 PMC545642

[B25] Fujita M Losick R Evidence that entry into sporulation in *Bacillus subtilis* is governed by a gradual increase in the level and activity of the master regulon Spo0A Genes Dev 2005 19 2236 2244 10.1101/gad.1335705 16166384 PMC1221893

[B26] Down JAT pH gradients in lepidopteran midgut J Exp Biol 1992 172 355 375 9874748 10.1242/jeb.172.1.355

[B27] Barboza-Corona JE Park HW Bideshi DK Federici BA The 60-kilodalton protein encoded by *orf2* in the *cry19A* operon of *Bacillus thuringiensis* subsp. *jegathesan* functions like a C-terminal crystallization domain Appl Environ Microbiol 2012 78 2005 2012 10.1128/AEM.06750-11 22247140 PMC3298145

[B28] Park HW Bideshi DK Wirth MC Johnson JJ Walton WE Federici BA Recombinant larvicidal bacteria with markedly improved efficacy against *Culex* vectors of West Nile virus Am J Trop Med Hyg 2005 72 732 738 15964958

[B29] Barboza-Corona JE Nieto-Mazzocco E Velázquez-Robledo R Salcedo-Hernández R Bautista M Jiménez B Ibarra JE Cloning, sequencing and expression of the chitinase gene *chiA74* from *Bacillus thuringiensis* Appl Environ Microbiol 2003 69 1023 1029 10.1128/AEM.69.2.1023-1029.2003 12571025 PMC143672

[B30] Bell RA Joachim FG Techniques for rearing laboratory colonies of tobacco hornworm and pink bollworms Ann Entomol Soc Am 1976 69 365 373

[B31] Ibarra JE Federici BA An alternative bioassay for determining the toxicity of suspended particles to mosquito larvae J Am Mosq Control Assoc 1987 3 187 192 3504908

